# Wanted Badly

**Published:** 1878-03

**Authors:** 


### WANTED BADLY.

A good many sentimental tears have been shed over that phantasy of an inspirited brain, "The song of a shirt." There is no doubt the writer had a glass of brandy, and a pipe beside him, when he wrote all that rigmarole about "stitch, stitch, stitch." There is nothing like "leather," but hard facts. Some time ago, a friend wanted a dozen shirts made, and asked our aid in finding a seamstress; attracted by a sign, a young woman presented herself; she was asked to name her price for making one; and was told that if the work was well done she could have the remainder at her own price. The article was dilatorily made, sent home and paid for; the bosom buttons came off before the man was dressed; on examination, it was found that they were attached by a single thread, and even that, loosely. Who ever purchased a ready made garment that did not want repairing after a week's use, either as to buttons or rips. We feelingly know that a really good dressmaker commands two dollars a day, coming at eight o'clock and leaving at six, wanting two very hearty meals, with tea at both, two or three cups of it, of the strongest kind, costing one dollar and a half a pound; and to get this same woman, even for a day, is sometimes the work of a month, that is, she generally is engaged that long before hand; to be sure she understands her business, and does it well. A common sewer demands a dollar a day and board; but seldom can be had without a fortnight's notice; there are hundreds of families in New York city to day, who would gladly pay high prices for a person who could sew well. There are five thousand families in New York city who would cheerfully give from fifteen to twenty dollars a month for a cleanly, capable, honest and economical cook, who had no relations to feed; who had no visitors, and who retired at ten o'clock. There are five thousand places for house girls who are fully competent to the duties of their department. It is scarcely possible to go into any mechanical office or shop where work is well done, and get it promptly done, because such men have more work than they can do. It is only the incompetent who fail to do well in New York.

